# Diffuse Intraductal Papillary Mucinous Neoplasia in Both Native and Transplant Pancreases Despite Normal Residual Pancreas in the Respective First-Degree Donor

**DOI:** 10.14309/crj.0000000000000841

**Published:** 2022-08-31

**Authors:** Ahmed Dirweesh, Stuart K. Amateau

**Affiliations:** 1 Division of Gastroenterology, Hepatology and Nutrition, University of Minnesota, MN

## CASE REPORT

**Figure 1.**
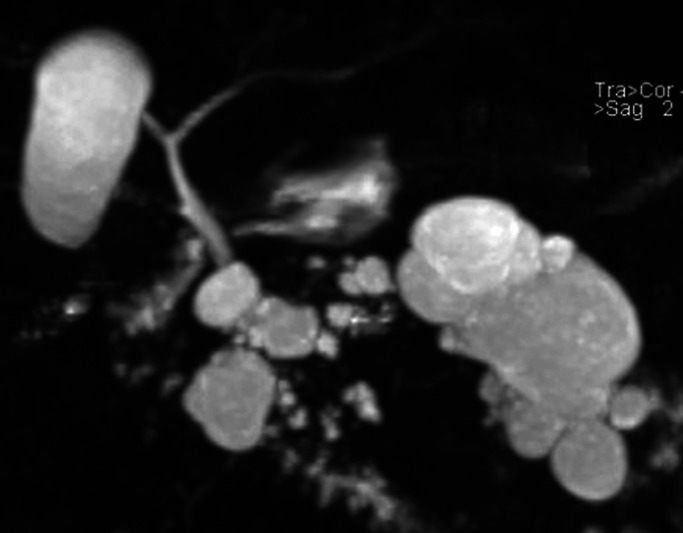
Magnetic resonance imaging with cholangiopancreatography (MRCP) reconstruction of the cystic transformation of the native pancreas.

**Figure 2.**
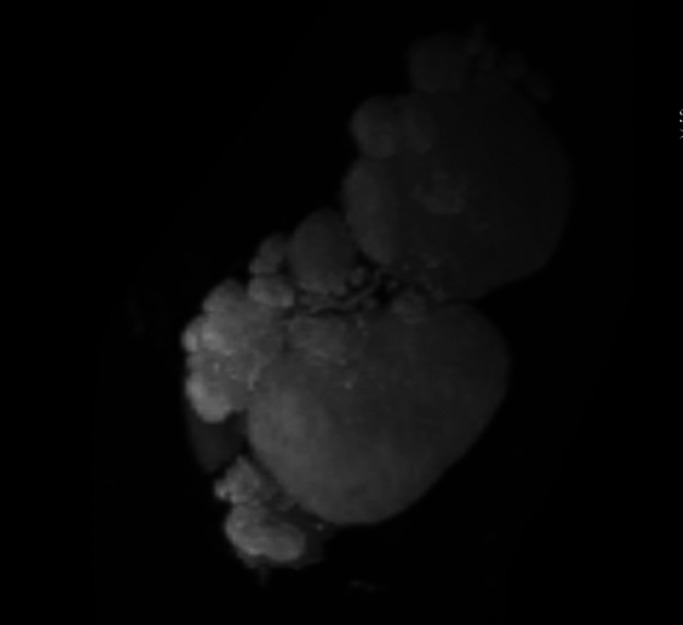
MRCP reconstruction of the cystic transformation of the transplant pancreas.

**Figure 3.**
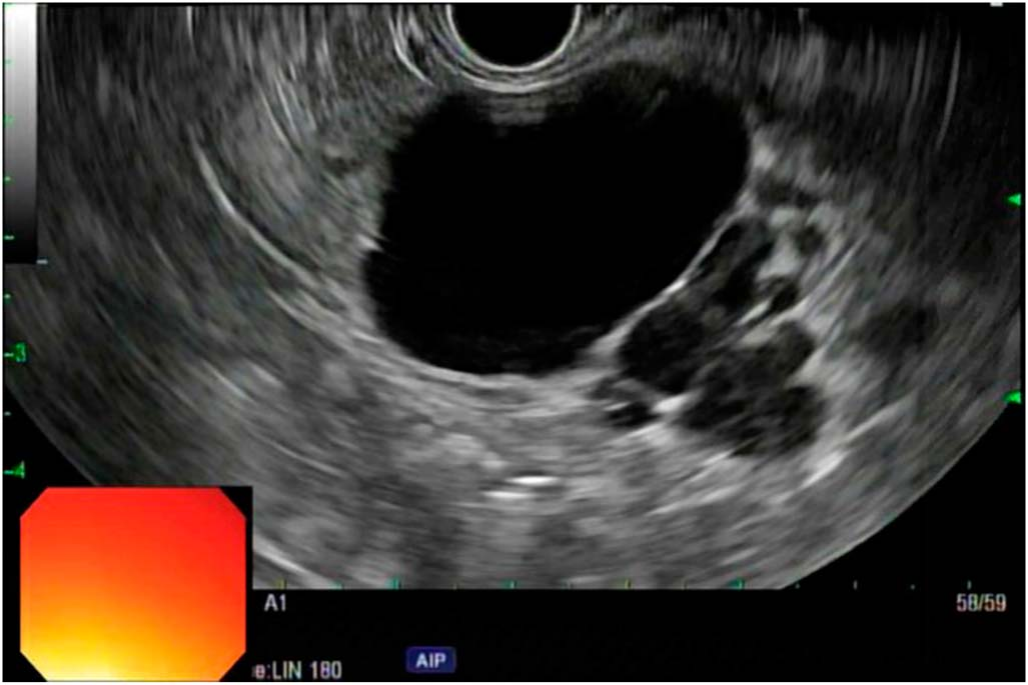
Endoscopic ultrasound (EUS) imaging of the cystic transformation of the native pancreas.

**Figure 4.**
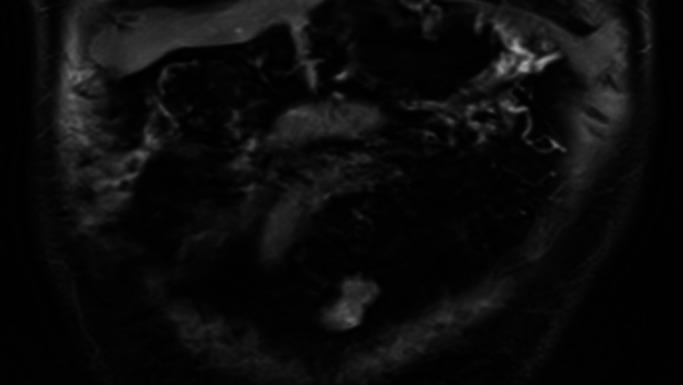
MRCP reconstruction of the normal residual first-degree donor's pancreas.

A 62-year-old man with a history of a simultaneous living donor pancreas-kidney transplant from his younger brother for end-stage diabetic nephropathy was found to have cystic replacement of both native and transplant pancreases on surveillance imaging. The patient was asymptomatic and without a family history of pancreatic disease. Magnetic resonance imaging with cholangiopancreatography revealed cystic lesions throughout native and transplant pancreases (Figures 1 and 2). Upper endoscopic ultrasound confirmed findings within the approachable native pancreas with cyst fluid aspirate showed intracellular mucin with high carcinoembryonic antigen and amylase. Subsequently, the first-degree donor underwent a screening magnetic resonance imaging with cholangiopancreatography revealing a normal remaining pancreas (Figures 3 and 4). Given the patient's comorbidities/poor operative candidacy and after a multidisciplinary discussion, invasive intervention was deferred in favor of interval imaging surveillance.

To the best of our knowledge, this is the first reported case of simultaneous intraductal papillary mucinous neoplasm of native and transplant pancreases, with the latter from a first-degree donor with unremarkable residual pancreas. This case illustrates that an immunocompromised recipient may develop significant cystic transformations in both organs, suggesting an environmental insult given lack of genetic/familial tendency.^1,2^ Management of patients with these de novo neoplasms, especially invasive ones, should consider their impaired immunity, comorbidities, and future risk of malignant transformation.^3^

## DISCLOSURES

Author contributions: AD Dirweesh and SK Amateau: Case concept, design, and drafting of the manuscript. SK. Amateau is the guarantor of the article.

Financial disclosure: None to report.

Potential competing interests: The authors declare that this work was conducted in the absence of commercial, financial, or nonfinancial relationships that could be interpreted as a potential conflict of interest. S.K.A is a consultant for Merit *Endoscopy*, Boston Scientific, US Endoscopy, Olympus medical, and Neurotronic and is the recipient of research support from Cook Medical.

Informed consent was obtained for this case report.
This manuscript has never been presented at a professional meeting.
